# Neural and Hybrid Modeling: An Alternative Route to Efficiently Predict the Behavior of Biotechnological Processes Aimed at Biofuels Obtainment

**DOI:** 10.1155/2014/303858

**Published:** 2014-01-05

**Authors:** Stefano Curcio, Alessandra Saraceno, Vincenza Calabrò, Gabriele Iorio

**Affiliations:** Laboratory of Transport Phenomena and Biotechnology, Department of Informatics, Modeling, Electronics and Systems Engineering, University of Calabria, Ponte P. Bucci, Cubo 39/C, 87036 Rende, Italy

## Abstract

The present paper was aimed at showing that advanced modeling techniques, based either on artificial neural networks or on hybrid systems, might efficiently predict the behavior of two biotechnological processes designed for the obtainment of second-generation biofuels from waste biomasses. In particular, the enzymatic transesterification of waste-oil glycerides, the key step for the obtainment of biodiesel, and the anaerobic digestion of agroindustry wastes to produce biogas were modeled. It was proved that the proposed modeling approaches provided very accurate predictions of systems behavior. Both neural network and hybrid modeling definitely represented a valid alternative to traditional theoretical models, especially when comprehensive knowledge of the metabolic pathways, of the true kinetic mechanisms, and of the transport phenomena involved in biotechnological processes was difficult to be achieved.

## 1. Introduction 

Mathematical modeling represents an effective support to the design, optimization, and control of biotechnological processes, which make use of enzymes or whole cells as catalysts. A comprehensive kinetic analysis of biocatalytic transformations, especially those ones aimed at the obtainment of second-generation biofuels from waste biomasses, is usually difficult to be achieved since many parallel-serial reactions are involved. In addition, the process efficiency may be strongly affected both by mass transfer limitations, which determine significant worsening of bioreactor performance, and by the presence of contaminants, which interfere with biocatalysts during the reaction progress.

Different approaches, that is, theoretical, empirical, semiempirical, were proposed in the literature to develop reliable models aimed at investigating how the responses of either biocatalytic processes or bioreactors change, with time, under the influence of both external disturbances and manipulated variables [1–4]. Fundamental or theoretical modeling is based on well-established conservation principles, whose exploitation allows formulating rather accurate kinetic/transport models describing the time evolutions of some characteristic parameters, namely, the bioreactor productivity or the substrate degree of conversion, as a function of the operating conditions [5]. An exhaustive analysis of all the complex phenomena occurring in a bioreactor, however, is difficult to be accomplished. The huge number of chemical reactions and a series of not-completely-understood phenomena related to the actual metabolic pathways involved in the process determine a significant level of uncertainness, which generally does not allow rigorous model formalization by proper mathematical relationships.

On the other hand, a model based on artificial neural networks (ANNs) does not make use of any kinetic or transport equation, which could help to determine, on the basis of fundamental principles, the mutual relationships existing between the inputs and the outputs [6]. ANNs are composed of interconnected computational elements, called neurons or nodes, which operate in parallel. Each neuron receives input signals from the related units, elaborates these stimuli by a transfer function, and generates an output signal, which, then, is transferred to other neurons belonging, in a forward configuration, to a succeeding layer. Even if the prediction of each single neuron could be imperfect and bias-affected, the outcome of the interconnection(s) among neurons is a reliable computational tool capable to learn from examples and to provide accurate predictions even with examples never seen before [7]. This feature makes ANNs a particularly useful tool when the behavior of complex systems is to be described, since no *a priori* knowledge of system dynamics is actually required. A neural model, however, can be rather complicated, since it may require a large number of connections and, therefore, a great number of parameters that are to be estimated. Generally, a larger number of neurons result not only in a more powerful network but also in a higher computational effort. The identification of the number of layers and of the neurons belonging to each layer is the result of an optimization process; although several methods were proposed to achieve the final network architecture, a general procedure is not yet available and the network structure is usually determined according to heuristic guidelines and to trial-and-error procedures [8–10]. The development of an artificial neural network model consists of several steps. During the training phase, the network learns how to correlate the input to the output variables. More specifically, the network is submitted to a certain number of input and output data, generally collected from experimental measurements; according to an error minimization algorithm, the weights characterizing each of the neurons are continuously updated. Only a certain number of the available experimental points are exploited during the training phase. The remaining data are used during a posttraining analysis, namely, the network test, during which the neural network is called to predict the output values corresponding to an input combination never seen before. The neural network test is performed in the definition domain in which ANN training was carried out. As a matter of fact, the forecasting capability of the neural networks outside this definition range cannot be guaranteed. Due to the intrinsic black-box nature of neural networks models, the validity domain does indeed strictly depend on the range of data used in model definition [11]. Another kind of posttraining analysis is the so-called validation phase during which the network is called to predict the experimental points excluded from both the training and the test sets.

A reasonable trade-off between theoretical and neural network approach is represented by hybrid neural modeling, leading to a so-called “grey-box” model capable of good performance in terms of data interpolation and extrapolation [13]. Hybrid neural model (HNM) predictions are given as a combination of both theoretical and “pure” neural network approach, together concurring at the obtainment of system responses. The main advantage of hybrid neural modeling regards the possibility of describing some well-assessed phenomena by means of a theoretical approach, leaving the analysis of other aspects, very difficult to interpret and describe in a fundamental way, to rather simple “cause-effect” models [14–16]. Two kinds of HNMs can be generally defined depending on the interactions existing between the neural and theoretical blocks. In a model based on a parallel architecture, the inaccuracy in the predicted value from the fundamental part is minimized by the addition of the residuals calculated by the neural network. In a model based on a serial architecture one (or more) process variable, which is difficult to measure, is estimated by a neural network and, then, fed to the theoretical block as an input. Finally, the outputs coming out from the fundamental part are checked with the experimental values for convergence.

In the present paper, two different biocatalytic processes aimed at the obtainment of second-generation biofuels from waste biomasses were modeled to show that either ANNs or HNMs could provide reliable predictions of the systems behavior. The attention was focused on waste biomasses since they are considered as one of the few current sustainable resources available for the production of renewable energy. The use of (bio)engineering and advanced modeling techniques was indeed believed as crucial to make the transition from a fossil fuel economy to a biomass-based economy a reality. In particular, a hybrid neural paradigm was exploited to model the kinetics of the enzymatic transesterification of waste-oil glycerides, actually the key step for the obtainment of biodiesel. An artificial neural network model was, instead, formulated to analyze the anaerobic codigestion of a mixture of agroindustry wastes aimed at biogas production; the neural model was also exploited to achieve the maximization of methane cumulative productivity as a function of mixture composition fed to the digester.

## 2. Description of the Considered Case Studies

Transesterification represents the alcoholysis of triglyceride esters resulting in a mixture of monoalkyl esters and glycerol. It is definitely the most widespread process on an industrial scale to convert vegetable oils into fuel form [17]. The general transesterification reaction scheme can be represented as [18]

(1) $$\begin{array}{c} \text{Triglyceride}\left(\text{TG}\right)+{\text{R}}^{\prime }\text{OH}⇄\text{Diglyceride}\left(\text{DG}\right)+{\text{R}}^{\prime }{\text{COOR}}_{1} \end{array}$$

(2) $$\begin{array}{c} \text{Diglyceride}\left(\text{DG}\right)+{\text{R}}^{\prime }\text{OH}⇄\text{Monoglyceride}\left(\text{MG}\right)+{\text{R}}^{\prime }{\text{COOR}}_{2} \end{array}$$

(3) $$\begin{array}{c} \text{Monoglyceride}\left(\text{MG}\right)+{\text{R}}^{\prime }\text{OH}⇄\text{Glycerol}\left(\text{GL}\right)+{\text{R}}^{\prime }{\text{COOR}}_{3} \end{array}$$

The high-viscosity compound glycerol is separated and removed so to achieve a low-viscosity final product similar to conventional diesel fuel; the mixture of resulting monoalkyl esters represents a good substitute for fossil fuels. The transesterification process can be performed in different ways, namely, by an alkaline catalyst, by an acid catalyst, or by a biocatalyst, immobilized in a proper support. The enzymatic process offers some advantages, such as a higher yield and a better glycerol recovery, as well as the possibility to use free fatty acid containing oils avoiding the formation of saponification products in the reaction mixture [19, 20]. Some additional advantages regard (a) the utilization of rather mild operating temperatures (up to 313 K) [21], (b) the reesterification of free fatty acids achieved by lipase [22, 23]; (c) the exploitation of alcohols containing some water (such a possibility is instead precluded in processes employing chemical catalysts) [24], and (d) the use of relatively simple downstream processing steps to purify biodiesel and by-products [25].

In the present paper, the enzymatic transesterification of triolein contained in waste olive oils was considered. The reaction pattern, as derived from the above (1)–(3), leads to the formation of one mole of ester, the ethyl oleate, for each of the three reactions and to the obtainment of glycerol only at the third step, when monoglycerides are actually converted:

(4) $$\begin{array}{c} \text{Triolein}+\text{Ethanol}⟷\text{Diolein}+\text{Ethyl}\;\text{Oleate} \end{array}$$

(5) $$\begin{array}{c} \text{Diolein}+\text{Ethanol}⟷\text{Monolein}\;+\text{Ethyl}\;\text{Oleate} \end{array}$$

(6) $$\begin{array}{c} \text{Monolein}+\text{Ethanol}⟷\text{Glycerol}+\text{Ethyl}\;\text{Oleate} \end{array}$$

Biogas can be produced from a variety of biomass feedstocks, including agricultural and livestock residues. Biogas is a versatile renewable energy source, which can be used to replace fossil fuels for power and heat production and as vehicle fuel [26]. Codigestion of mixed substrates offers many advantages, including ecological, technological, and economic benefits, compared to digesting a single substrate [27]. The purpose of codigestion is to balance nutrients (C/N ratio and macro- and micronutrients) and dilute inhibitors/toxic compounds to enhance methane production. However, combining two or more different types of feedstocks requires careful selection of biomasses main characteristics to improve the efficiency of anaerobic digestion. The performance in terms of biogas production and digestate quality depends on several parameters such us temperature, organic load rate (OLR), hydraulic retention time (HRT), and feedstock composition [28]. A cost effective way to facilitate future development of agroeconomy is represented by the replacement of energy crops with several kinds of agrowaste in anaerobic digestion plant feedstocks [29].

In the present paper, the anaerobic codigestion process of two wastes, namely, a mixture of manure and orange juice waste (OJW), was analyzed. It was intended to propose an advanced modeling approach aimed at predicting the behavior of an anaerobic digester and at identifying a proper feed strategy of the considered two substrates so as to maximize methane productivity. Due to the complexity of anaerobic digestion process, a pure ANN model was exploited.

## 3. Materials and Methods

Two experimental protocols were set up to collect the experimental data necessary to develop the present HNM and ANN models aimed at characterizing the transesterification of glycerides and the anaerobic codigestion process, respectively.

### 3.1. Transesterification of Glycerides

The experimental runs were performed using a simulating oil, having a 60% (w/w) of pure triolein; the remaining 40% of the mixture included fatty acid or mono- and diglycerides. Ethanol (99.8% grade) from Fluka was used as the secondary substrate; hexane (95% grade) from Fluka was the solvent, as suggested in the literature [30]. Distilled water was exploited to perform the tests not in anhydrous conditions.

The biocatalyst was Lipozyme MM IM (Novozymes, Denmark), a lipase from *Mucor miehei* immobilized on a macroporous ion exchange resin. The diameter of the supporting particles ranged between 0.3 and 1.0 mm and their wet bulk density was 0.42 g/mL. The enzyme was highly 1,3 specific, with a molecular weight of 32 KDa and an activity of 37 U/g. All the experiments were performed at an operating temperature of 37°C and neutral pH by a well-mixed batch reactor having a volume of 125 mL. The batch runs were designed varying the following variables: (1) the mass feed ratio of enzyme/triolein (e_0_/t_0_), (2) the reactants molar ratios of ethanol/triolein (Et_0_/T_0_), (3) the mass of water fed to the bioreactor (W_0_), (4) the stirring rate (*ω*) with three characteristic levels (0-1-2), and (5) the mass feed ratio (triolein/hexane) (T_0_/Hex_0_). The reaction mixture was prepared according to the procedure reported in [12]. Reaction samples of 200 *μ*L were collected, ensuring not to have any catalyst in the sample and avoiding that the total amount of collected samples was 5% greater than the total volume. The operating conditions of all the performed experimental runs were summarized in Table 1.

Concentrations of reactants, for example, glycerides, and product, that is, ethyl oleate, were quantitatively measured by high performance liquid chromatography, HPLC (JASCO), under the following conditions: RI detector, eluent phase composition: acetone/acetonitrile 70/30 v/v (HPLC grade, Fluka), flow rate 1 mL/min, and internal normalization as integration method. Prior to each analysis, both the catalyst and the hexane were removed by centrifugation and by evaporation, respectively. Ethanol concentrations were not directly measured but obtained assuming a 1 : 1 stoichiometric ratio with ethyl oleate. The utilized HLPC column was Alltech Adsorbosphere HS (C18) 5 *μ*m, having a length of 250 mm and an inlet diameter of 4.6 mm; the column was provided with a 7.5 × 4.6 mm Alltech precolumn.

### 3.2. Anaerobic Codigestion of Agroindustry Wastes

The anaerobic codigestion process was performed in a pilot-scale batch reactor. The reactor had a volume of 23.7 l and was filled with 15 l of manure/OJW mixture; the remaining reactor volume was intended for the produced biogas. The experimental runs operating conditions were designed so to test five different manure/OJW feed ratios, on a mass basis (Table 2). The charged matrix was characterized in terms of the initial chemical oxygen demand (COD_0_), ranging from 95 to 102 g/L, the initial pH (pH_0_), ranging from 4.5 to 6.8, and the initial carbon/nitrogen ratio (C/N)_0_, ranging from 27.8 to 29.6. Each reaction run had a total duration of 28 days and was performed at a constant temperature of 38°C.

The reaction progress was followed by measuring the composition of the produced biogas (Agilent gas-chromatograph) and both COD and pH of the mixture contained in the digester. It is worthwhile remarking that, due to the complex reaction pathways involved in the anaerobic digestion of real agroindustry wastes, it was not possible to identify a single substrate whose concentration could be directly related to methane production. As a consequence, the COD measurement was considered as an inferential measurement of the overall substrates concentration.

## 4. Model Development

### 4.1. Enzymatic Transesterification of Olive Oil Glycerides

The reaction pattern of biocatalytic transesterification of triolein in the presence of ethanol was already analyzed [12]. The complex kinetic mechanism was actually described by a Ping-Pong Bi-Bi mechanism with ethanol inhibition; the King-Altman kinetics method, based on singling out geometrical rules that permitted evaluating the concentrations of enzyme in all its complexes ([E], [e], [ES], [EP], etc.), was also adopted. By considering the actual rate of each elementary reaction, it was possible to formulate the overall kinetic rate equation, expressed as the disappearance of triolein [T], as follows:

(7) −d[T]dt =((K1[T][Et]−K2[P][EO])    ×(K3[T]+K4[Et]+K5[T][Et]+K6[P]      +K7[EO]+K8[P][EO]+K9[T][P]      +K10[Et][EO]+K11[Et]2+K12[Et][P])−1)·[e0],

where [T] represented triolein concentration (mol/L); [Et] was ethanol concentration (mol/L); [P] was the overall concentration of glycerol, monolein, and diolein (mol/ L); [EO] was ethyl oleate concentration (mol/L) and [e_0_] was lipase concentration (g/L);  *K*_*i*_  (*i* = 1,…, 12)  were some model parameters, strictly related to the reactions kinetic constants.

On the basis of stoichiometry and some semiempirical correlations obtained analyzing the collected experimental data, the concentrations of products and ethanol were expressed [12] as a function of triolein actual concentration [T] and substrates initial concentrations [T_0_] and [Et_0_]:

(8a) $$\begin{array}{c} \left[\text{Et}\right]=2.25\cdot \left(\left[\text{T}\right]-\left[{\text{T}}_{0}\right]\right)+\left[\text{E}{\text{t}}_{0}\right] \end{array}$$

(8b) $$\begin{array}{c} \left[\text{EO}\right]=-2.25\cdot \left(\left[\text{T}\right]-\left[{\text{T}}_{0}\right]\right) \end{array}$$

(8c) $$\begin{array}{c} \left[\text{P}\right]=\left[{\text{T}}_{0}\right]-\left[\text{T}\right] \end{array}$$

In the present paper, a completely different methodology aimed at determining the actual relationship existing among the concentrations of products and ethanol and the variables [T], [T_0_], and [Et_0_] was presented. The already-determined linear relationship between triolein and ethyl oleate, in principle, might not be accurately verified in some cases, especially when the concentrations of reactant(s) or of product(s) are low, that is, at the beginning or at the end of the reaction. Product obtainment, in fact, exhibited in some circumstances an initial delay with reference to substrate consumption; a final decrease of product production rate as compared to substrate consumption rate was observed as well. An improper estimation of the actual substrate(s)-product(s) relationship, therefore, could lead to unfair predictions of the biocatalytic reaction under study, especially if it is considered that initial rate strongly affected the actual process dynamics, whereas the final values were critical when reaction yield and substrate conversion were to be calculated. The exploited methodology made use of advanced computational models, based on artificial neural networks, properly integrated with the already-proposed kinetic mechanism so as to formulate an overall hybrid neural model (HNM), which was expected to provide more reliable predictions of the actual time-evolutions of substrate(s) and product(s) concentrations involved in the biocatalytic transesterification process. Therefore, the reaction mechanism and the relative kinetic equation were determined using a rigorous theoretical approach whereas the relationship existing between substrate and product concentrations was determined using an ANN, hereafter called ANN_1_. This was characterized by a single output variable, the ethyl oleate formation, expressed in terms of the concentration difference ([EO(*t*)]−[EO_0_]). A set of input variables was considered as significant on the basis of a sensitivity analysis performed on the biocatalytic process under study. Such variables, which exhibited the highest influence on product formation, were (a) the enzyme/triolein initial mass ratio ([e_0_]/[t_0_]), (b) the ethanol/triolein initial molar ratio ([Et_0_]/[T_0_]), (c) the initial water content of the reaction mixture ([W_0_]), (d) the reactor agitation rate (rpm), (e) the triolein/hexane initial ratio ([T_0_]/[Hex_0_]), and (f) the triolein consumption, expressed in terms of concentration difference ([T_0_]−[T(*t*)]).

**Figure 1:**
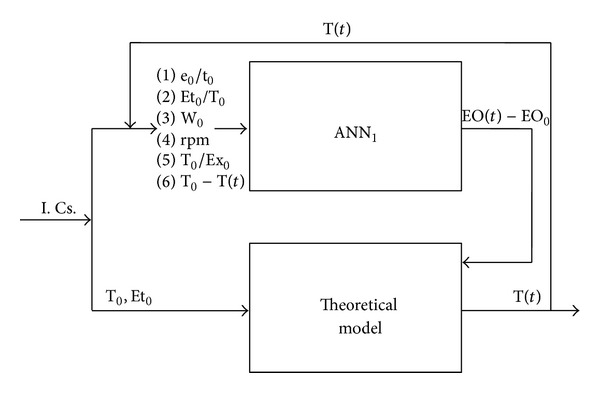
Input-output structure of the developed hybrid neural model.

The input-output structure of the developed hybrid neural model was schematized in Figure 1.

The HNM had a parallel architecture, characterized by the continuous transfer of information between the theoretical and neural parts of the model. In order to develop the neural model, the available experimental data, corresponding to 144 points, were randomly split into three groups, reserving 70 points to network training and 24 points to test the neural network predictions. The remaining 50 points were used to validate the predictions of the developed ANN in a set of conditions never exploited neither during learning nor during network test. A multilayer perceptron (MLP) feed-forward architecture with a pyramidal structure having a decreasing number of neurons from the input to the output layer was identified by Matlab Neural Network Toolbox (The Mathworks), Version 4.0.1, according to the trial-and-error procedure described in [31]. The method adopted to improve neural network generalization was the so-called Bayesian regularization, which assumes that the weights and the biases of the network are random variables with specified distributions. The resulting structure of ANN_1_ consisted of an input layer with 6 neurons (corresponding to the input variables), a first hidden layer with 10 neurons, a second hidden layer with 3 neurons, and a single-neuron output layer. The neurons transfer function characterizing input and hidden layers was the hyperbolic tangent; the single-neuron output layer was instead characterized by a linear transfer function. It is worthwhile observing that the developed network had to account for the effects of a considerable number of inputs on process performance; this definitely required a larger number of connections and, therefore, a larger number of parameters with respect to a “traditional” one-hidden-layer neural architecture.

### 4.2. Anaerobic Digestion of Agroindustry Wastes

As mentioned in the previous section, due to the complexity of agroindustry wastes, anaerobic codigestion, a pure neural model, hereafter called ANN_2_, was developed to predict the time evolution of the digester and, then, to analyze the effect of a variation of feed mixture composition on process performance. Methane cumulative productivity was chosen as ANN_2_ output variable. The input variables were: (a) the OJW mass percentage; (b) the values of (b1) pH (pH_0_), (b2) chemical oxygen demand (COD_0_), and (b3) carbon-nitrogen ratio ((C/N)_0_) of the mixture fed to the digester at the beginning of each batch run; (c) the process time, *t*, ranging from 0 to 28 days. The available experimental data, corresponding to 140 points, were split into three groups, reserving 70 points to network training and 21 points to test the neural network predictions. The remaining 49 points (35% of the total) were used to validate the predictions of the developed ANN_2_; all the experimental results corresponding to run 3 of Table 2 were included in the validation dataset and were used to validate the developed neural model predictions in a set of conditions never exploited neither during learning nor during test. As in the previous case, a multilayer perceptron (MLP) feed-forward architecture, developed on the basis of a Bayesian learning algorithm, was eventually identified by Matlab Neural Network Toolbox. The resulting structure of ANN_2_ consisted of an input layer with 5 neurons, a first hidden layer with 6 neurons, a second hidden layer with 2 neurons, and a single-neuron output layer. The neurons transfer functions were the hyperbolic tangent, for input and hidden layers and the linear transfer function for the output layer. The developed ANN_2_ model was exploited to estimate the effect of feed mixture composition on anaerobic codigestion performance; this allowed identifying a feeding strategy, which improved the cumulative concentration of methane in the obtained biogas. The procedure exploited to improve the digester performance consisted of the following steps: (1) the batch time, that is, 28 days, was subdivided into three periods: (a) days 1–9, (b) days 10–19, and (c) days 20–28; (2) at the beginning of each of the periods, it was assumed that OJW fraction in the feed mixture could be equal to either 0% or 10% or 15% or 20% (on a mass basis); (3) a grid of possible reaction progresses, resulting in 64 different scenarios, was obtained on the basis of the different OJW mass fractions set at the beginning of each time period; (4) methane cumulative productivity was calculated by ANN_2_ for each of the obtained scenarios in order to determine the feeding strategy, which permitted improving methane production and, therefore, the fermenter performance.

## 5. Results and Discussions

**Figure 2:**
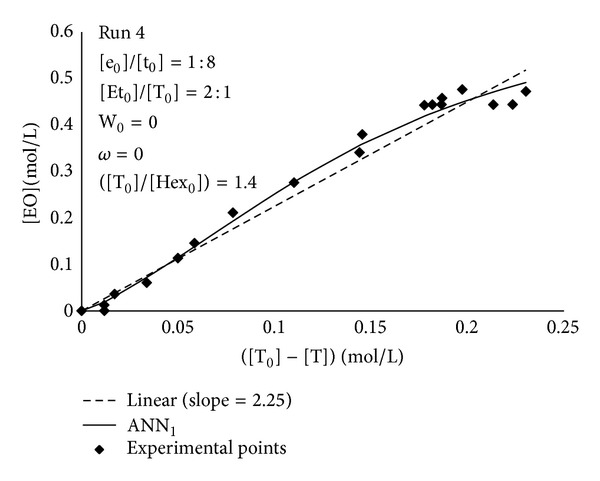
Comparison between experimental data, ANN_1_ prediction and linear empirical correlation ([EO] = 2.25∗([T_0_]−[T]) exploited in the previous paper [12], for the determination of the relationship between ethyl oleate production and triolein consumption. Operating conditions: run 4 of Table 1.

Figure 2 showed a comparison between the experimental points and the predictions provided by ANN_1_ in a typical case. It is worthwhile observing that the experimental data of produced ethyl oleate ([EO(*t*)]−[EO_0_] = [EO]) versus triolein consumption ([T_0_]−[T]) did not show a linear trend, as it was supposed in the previous paper [12]. On the contrary, a sigmoid-like trend could be recognized.

**Figure 3:**
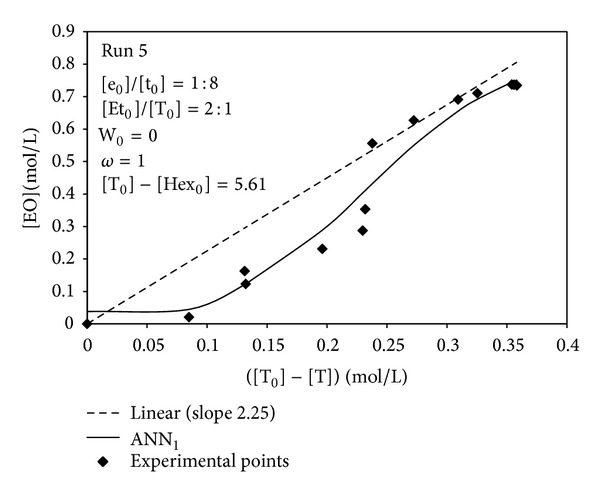
Comparison between experimental data, ANN_1_ prediction, and linear empirical correlation ([EO] = 2.25∗([T_0_]−[T]) exploited in [12], for the determination of the relationship between ethyl oleate production and triolein consumption. Operating conditions: run 5 of Table 1.

Similar trends were observed under different operating conditions (Figure 3) and for all the experiments whose operating conditions were summarized in Table 1 (data not shown), although the deviations from the linear trend were, in some cases, less pronounced than those presented in Figures 2 and 3.

**Figure 4:**
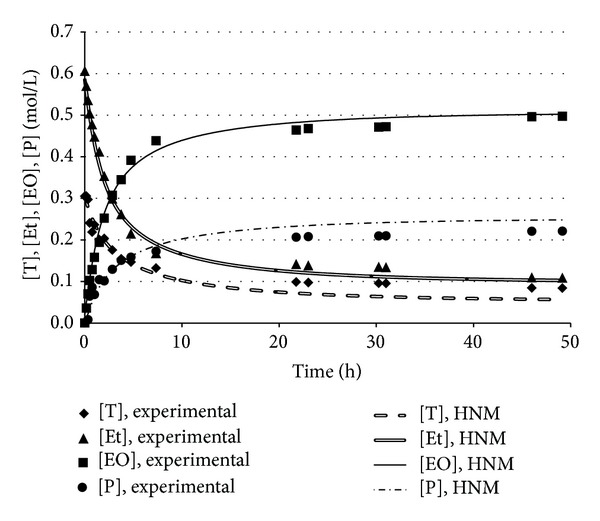
Comparison between the experimental data and the concentrations predicted by the hybrid neural model. Operating conditions: run 6 of Table 1.

**Figure 5:**
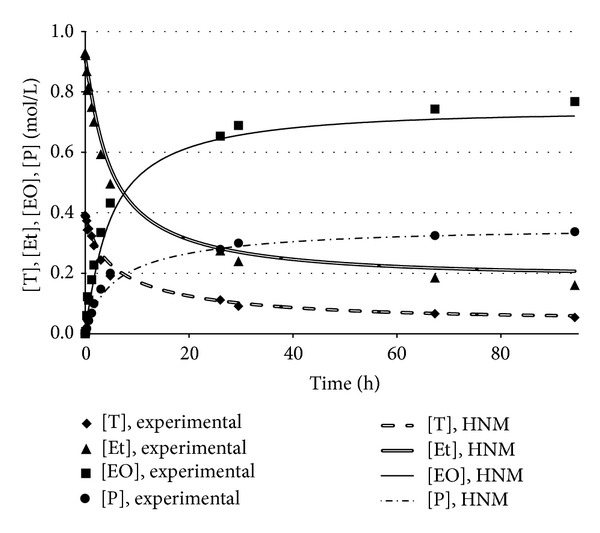
Comparison between the experimental data and the concentrations predicted by the hybrid neural model. Operating conditions: run 7 of Table 1.

The developed neural model ANN_1_, therefore, predicted more reliably than a linear correlation the actual relationship between ethyl oleate and triolein concentrations. ANN_1_ predictions were exploited by the formulated hybrid neural model to calculate the time evolutions of the concentrations of all the reacting species participating in the transesterification reaction. Figures 4 and 5 reported the experimental data and the corresponding simulation results obtained by the developed hybrid neural model in two different operating conditions, corresponding to runs 6 and 7, respectively. A remarkable agreement between the experimental points and the simulation results was observed; HNM was capable of reliably predicting both the time variation and the plateau values for either reaction products, [EO] and [P], or substrates, [T] and [Et].

Similar good agreements were obtained in all the other experimental conditions summarized in Table 1 (data not shown). On the basis of the obtained results, it could be concluded that the developed hybrid neural model represented an effective computational tool capable of predicting the actual system behavior over a wide range of process and operating conditions.

**Figure 6:**
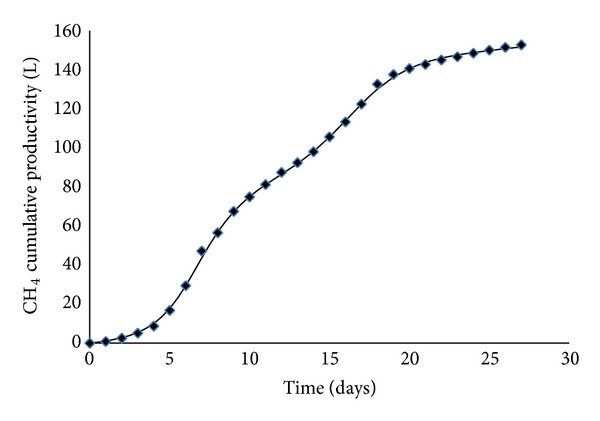
Comparison between experimental data (belonging to the test/training dataset) and methane cumulative productivity as predicted by the ANN_2_. Operating conditions: run 4 of Table 2.

**Figure 7:**
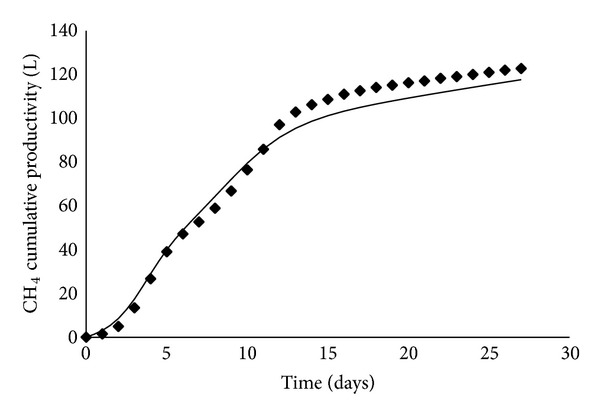
Comparison between experimental data (belonging to validation dataset) and methane cumulative productivity as predicted by the ANN_2_. Operating conditions: run 3 of Table 2.

As far as the anaerobic codigestion process was concerned, the performance of the developed neural model was evaluated comparing the predictions provided by ANN_2_ with the experimental results collected during pilot digester operations. Figures 6 and 7 showed, in two typical cases, the calculated time evolutions of methane cumulative productivity, expressed as the liters of produced methane in standard conditions, and the corresponding experimental points. Actually, a very good agreement could be observed, when the experimental data belonged to either training/test (Figure 6) or validation (Figure 7) dataset; the model was indeed capable of reliably reproducing the experimental measurements since the maximum relative error never exceeded 6%.

**Figure 8:**
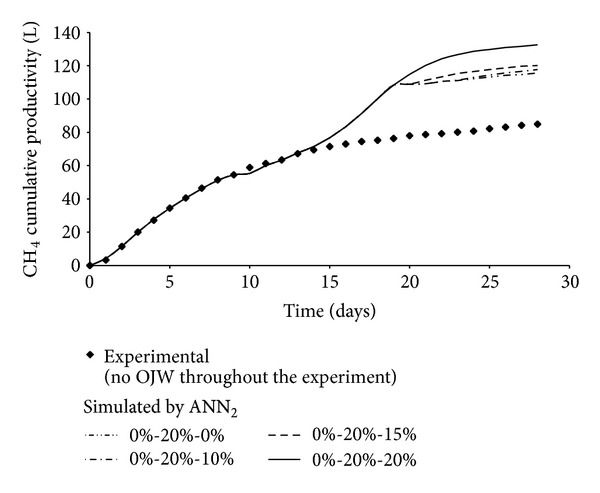
Effect of the variation of feed mixture composition on methane cumulative productivity.

The above results suggested the possibility of analyzing the effect of feed mixture composition on fermenter performance; in particular, it was intended to improve methane productivity by properly programming the variation of feed mixture composition. As already mentioned in the previous section, all the identified possible scenarios were simulated by ANN_2_.  Figure 8 showed, in some typical cases, that a variation of feed mixture composition was responsible, as compared to the experimental points measured when feed mixture had a constant composition (only manure), for an improvement of process performance. With reference to Figure 8, it could be observed that if the digester was started up with manure only and, after 9 days of operation, the OJW percentage in the feed was increased to 20% and, then, kept constant, the best performance was obtained. The developed neural model, therefore, represented a valuable computational tool, which provided useful indications about anaerobic codigestion process.

## 6. Conclusions

In the present paper, it was shown that both the hybrid neural approach and the pure neural model turned out to be very efficient tools for the analysis and simulation of two biotechnological processes, which could be very difficult to interpret in a traditional way. The developed models permitted to overcome the difficulties involved in the theoretical description of the complex reaction mechanisms characterizing the obtainment of second-generation biofuels (biodiesel and biogas) from waste biomasses.

The observed reliability of models predictions suggests the possibility of developing and implementing a neural network predictive controller aimed at accomplishing proper control actions, which can be undertaken to optimize processes performance. Work is currently in progress for the realization of such a controller, as well as for the improvement of both the models features by a more precise description of the phenomena occurring in the considered reactors.

## Figures and Tables

**Table 1:** Experimental conditions exploited to perform the enzymatic transesterification reactions.

Run *N*°	e_0_/t_0_ [g/g]	Et_0_/T_0_ [mol/mol]	W_0_ [g/L]	*ω* [level]	T_0_/Hex_0_ [g/g]
1	1 : 8	2 : 1	1.0	1	1.4
2	1 : 8	2 : 1	0	1	1.4
3	1 : 8	2 : 1	0	2	1.4
4	1 : 8	2 : 1	0	0	1.4
5	1 : 8	2 : 1	0	1	5.61
6	1 : 20	2 : 1	0	1	1.4
7	1 : 20	2.5 : 1	0	1	1.4
8	1 : 20	3 : 1	0	1	1.4
9	1 : 4	2 : 1	0	1	0.69

**Table 2:** Manure/OJW ratios exploited to perform the anaerobic digestion of agroindustry wastes.

Run *N*°	Manure (mass %)	OJW (mass %)
1	100	0
2	95	5
3	90	10
4	85	15
5	50	50
